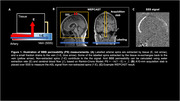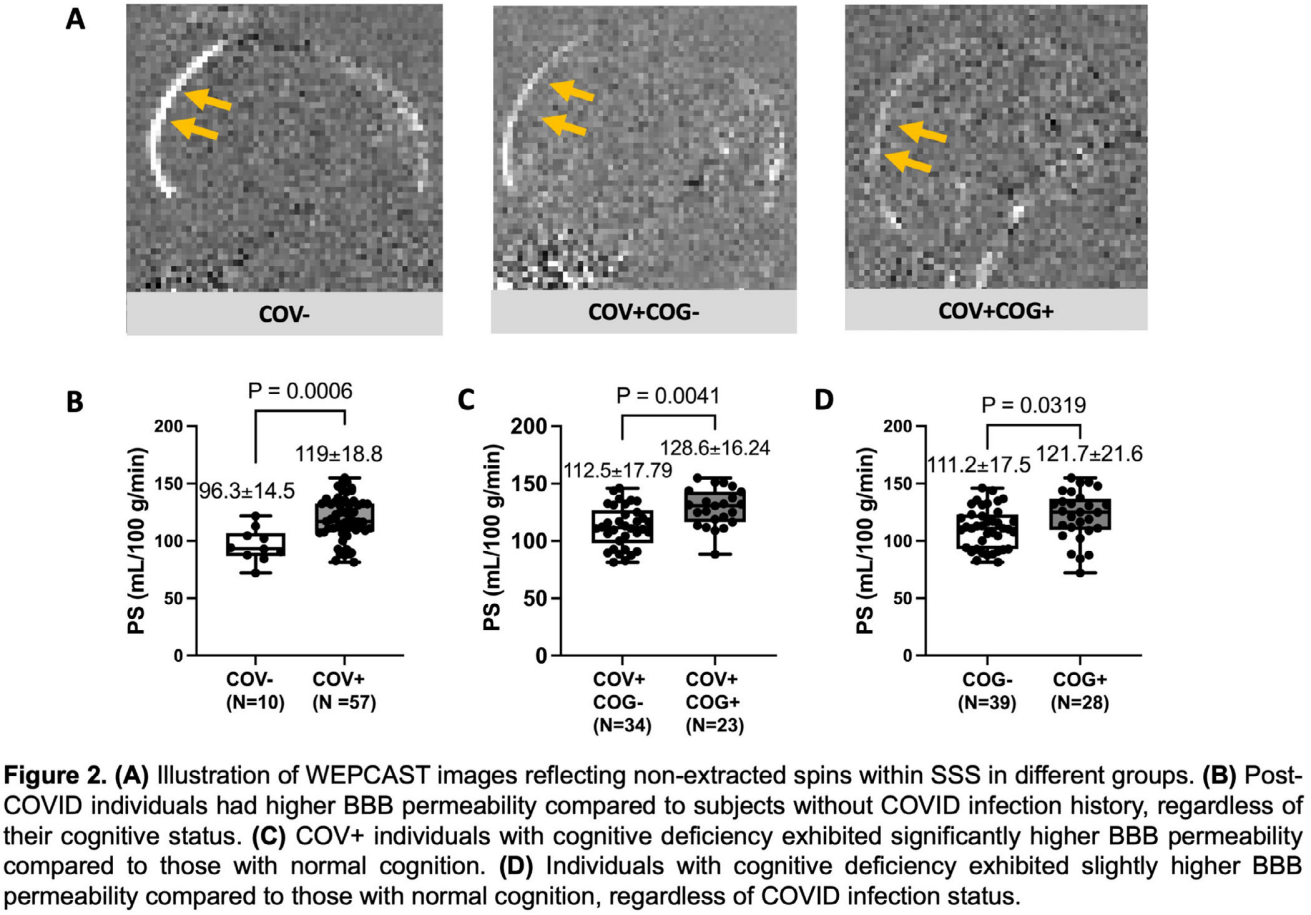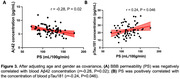# Increased blood‐brain barrier permeability following COVID‐19 and its correlation with Alzheimer’s biomarkers

**DOI:** 10.1002/alz.086505

**Published:** 2025-01-09

**Authors:** Zhe Sun, Jennifer Frontera, Arjun V. Masurkar, Allal Boutajangout, Li Jiang, Sujata Thawani, Jon Links, Alok Vedvyas, Rebecca A Betensky, Ludovic Debure, Wajiha Ahmed, Thomas Wisniewski, Yulin Ge

**Affiliations:** ^1^ NYU Grossman School of Medicine, New York, NY USA; ^2^ Alzheimer's Disease Research Center, New York University Langone Health, New York, NY USA; ^3^ NYU Alzheimer's Disease Research Center, New York, NY USA; ^4^ New York University Grossman School of Medicine, New York, NY USA

## Abstract

**Background:**

Post‐COVID cognitive dysfunctions, impacting attention, memory, and learning, might be linked to inflammation‐induced blood‐brain barrier (BBB) impairment. This study explores post‐COVID BBB permeability changes using a non‐contrast water‐exchange based MRI and their associations with blood Alzheimer’s biomarkers.

**Method:**

Sixty‐seven participants were classified based on COVID (COV) and cognitive (COG) statuses into three groups: COV+/COG‐ (n=34), COV+/COG+ (n=23), and COV‐ (n=10) for comparisons (COV+: Laboratory‐verified SARS‐CoV‐2 infection; COV‐: No history of SARS‐CoV‐2 infection and negative SARS‐CoV‐2 nucleocapsid antibody test.). All patients underwent neuropsychological testing and were coded as having cognitive impairment (COG+) based on neuropsychological testing (≥1 abnormal test result). Clinician interview with the patient and informant. Patients were imaged with the following 3T‐MRI sequences: 1) T1‐MPRAGE; 2) Phase contrast (PC)‐MRI for quantification of cerebral blood flow (ƒ). 3) Water‐extraction‐with‐phase‐contrast‐arterial‐spin‐tagging (WEPCAST) [1], estimating permeability‐surface‐area‐product (PS) calculated with Renkin‐Crone Model: PS = ‐ln (1‐E) ∗ƒ (Figure 1). Plasma biomarkers of Alzheimer’s disease (AD), including Aβ40, Aβ42, NFL, and pTau181, were measured on the same day as the MRI. PS was compared between groups. Correlations were computed between PS and AD biomarkers.

**Result:**

We included 67 non‐hospitalized participants (age: 64.7, F/M: 43/24) who successfully completed their clinical and MRI exams. Illustrative WEPCAST images and the results of group comparisons indicated that BBB permeability (PS) was higher in COV+ than that in COV‐ subjects (119 vs 96 mg/100 g/min, P=0.006), as well as in any patient with cognitive dysfunction (COG+ vs COG‐, 122 vs 111 mg/100 g/min, P=0.0319). Similarly, among COV+ subjects, PS was significantly higher in COV+COG+ compared to COV+COG‐ subjects (129 vs 113 mg/100 g/min, P=0.0041; Figure 2). After adjusting age, PS was negatively correlated with plasma Aβ42 (r=‐0.28, P=0.02), and positively correlated with pTau181 (r=0.24, P=0.046) in all subjects. (Figure 3). There was no significant correlation between PS and NLF or Aβ40.

**Conclusion:**

Our findings demonstrate that SARS‐CoV‐2 virus has an impact on BBB integrity. This effect correlates with several AD biomarkers, including plasma Aβ42 and pTau181, highlighting the potential contribution of increased BBB permeability in post‐COVID to AD pathology.

**Reference**: 1. Lin Z, et al. Magn. Reson. Med. 2018;80:1507–1520.